# Heterogeneous and Composite Bioinks for 3D-Bioprinting of Complex Tissue

**DOI:** 10.1007/s44174-024-00171-7

**Published:** 2024-03-29

**Authors:** Rahimeh Rasouli, Crystal Sweeney, John P. Frampton

**Affiliations:** 1 School of Biomedical Engineering, Dalhousie University, Halifax, Canada; 2 Department of Biochemistry and Molecular Biology, Dalhousie University, Halifax, Canada

**Keywords:** Bioink, 3D-bioprinting, Tissue engineering, Regenerative medicine, Hydrogel, Nanomaterial, Particle, Stimuli responsiveness

## Abstract

Bioink composition is a key consideration for the 3D-bioprinting of complex and stable structures used to model tissues and as tissue constructs for regenerative medicine. An emerging and industrially important area of research is the use of micro- and nanofillers to improve bioink performance without dramatically altering the physicochemical properties of the polymeric material that forms the bulk of the printed structure. The purpose of this review is to provide a comprehensive overview of emerging nanomaterial fillers designed to create heterogeneous and composite bioinks for 3D-bioprinting of complex functional tissues. We outline the criteria that must be considered when developing such a bioink and discuss applications where the fillers impart stimuli responsiveness, e.g., when exposed to magnetic fields, electrical fields, and light. We further highlight how the use of such fillers can enable non-destructive imaging to monitor scaffold placement and integrity following implantation.

## Introduction

**Fig. 1:**
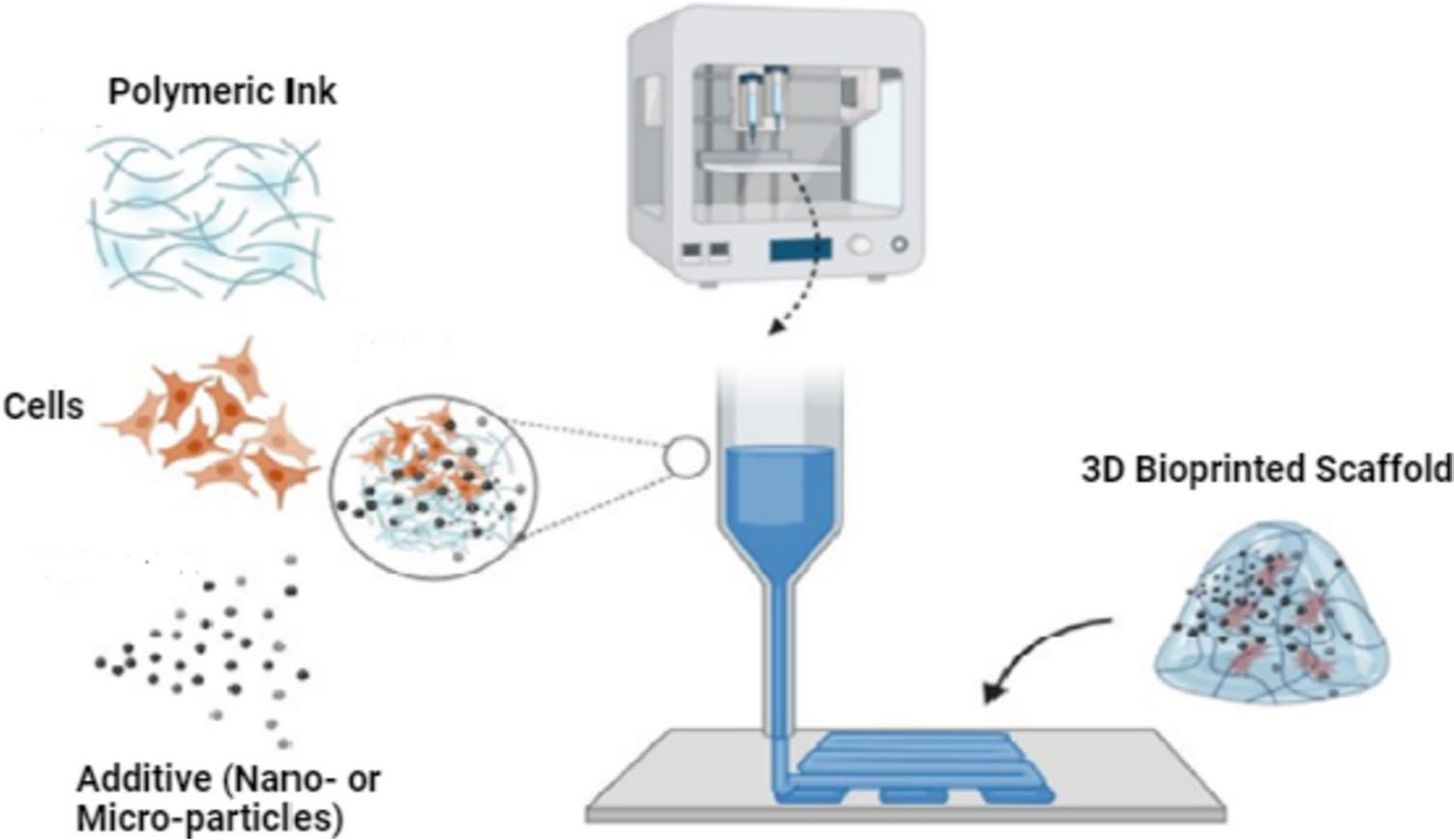
Schematic illustration of a typical 3D-bioprinting process. Figure created using BioRender. Other materials that may be added to bioinks include drugs, ions, growth factors, and various other bioactive molecules

Advancements in the biomanufacturing of complex tissues create new avenues in replacing and regenerating tissues and organs. Fully-functional complex tissues are paramount in addressing the organ shortage, repairing and reconstructing damaged tissues/tissue defects [1], and investigating pharmacological and toxicological effects of a plethora of therapeutics and chemicals in patient-specific disease models [2, 3]. 3D-bioprinting has opened many new possibilities for creating complex structures by way of precise deposition of a bioink composed of a formulated mixture of polymeric materials, biological materials such as cells, and in some cases, bioactive molecules and particulates [4, 5]. A typical 3D-bioprinting process generates a three-dimensional bioprinted scaffold by dispensing a suitable bioink in a layer-by-layer fashion using a digital file as a blueprint (Fig. 1). An ideal bioink intended for use in tissue engineering and regenerative medicine should (i) be cytocompatible; (ii) promote cell attachment; (iii) have appropriate viscosity and favorable gelation kinetics to permit extrusion; (iv) form scaffolds with sufficient mechanical strength, rigidity, and shape fidelity to hold shape and recapitulate the architecture and mechanics of the native cellular microenvironment; (v) be amenable to chemical alterations aimed to optimize the cellular microenvironment; and (vi) be scalable for industrial production with sufficient batch-to-batch precision [6]. However, there are currently few bioinks that are commercially available, because the ability to promote cell attachment and growth often competes with the fluid properties required for printing and the mechanical properties of the final printed structure required for handling and wear resistance. As such, most printing systems have been designed and optimized for simple bioinks formulated from relatively low-cost and abundant biomaterials, such as alginate, collagen, and gelatin, even though one can find examples of more complex bioink formulations that boast superior performance characteristics. An emerging clinically and industrially important area of research is the use of fillers to improve the bioink performance without dramatically altering the physicochemical properties of the polymeric material that must be crosslinked or gelled to form a stable print.

Bioinks can be composed of natural materials (e.g., alginate, gelatin, collagen, silk, and agarose), synthetic materials (e.g., polycaprolactone, polyethylene glycol, and pluronic), or a combination of both [7]. Composite bioinks are blends or multi-phase materials composed of two or more polymers with integrated bioactive inorganic fillers and encapsulated cells [8]. Bioactive molecules/particles that can be incorporated into bioinks include growth factors, blood plasma, proteins, peptides, and microbes (e.g., yeast [9], microalgae [10], and bacteria [11]) [7]. Heterogeneous bioinks are characterized by multiple cell types and additives that function collectively to mimic the target tissue or provide emergent function [12]. As the target environment of the 3D-bioprinted material is a complex biological system (often within the human body), the bioink must be composed of suitable materials that enable printing of complex and stable geometries [8]. The development of heterogeneous bioinks that incorporate fillers is a promising approach to increasing complexity and overcoming limitations of simple bioink formulations. For example, the incorporation of nanomaterial fillers such as hydroxyapatite (HAp), an osteoinductive agent, into bioinks enables high-resolution printing of structures with supportive functions for bone growth and osseointegration capable of replicating native tissues while further enhancing regeneration and therapeutic outcomes [7].

The development of a new generation of bioinks incorporating fillers such as the example above is paramount to achieving high print fidelity, maintaining shear-thinning characteristics essential for printing, and providing mechanical strength that matches the native tissue, improved cytocompatibility and biocompatibility, and the ability to modulate cell–matrix interactions. An additional consideration is control over the degradation rate and swelling of the printed scaffold. Printed scaffolds can undergo degradation through different mechanisms, such as enzymatic, hydrolytic, and ion exchange degradation based on the polymers and additives that are selected. The biodegradation kinetics can affect cell-mediated construct remodeling and extracellular matrix (ECM) production. Therefore, the degradation kinetics of the polymerics scaffold and additives in response to cell–matrix remodeling is an essential consideration for bioink design that has not been well explored for structures formed using composite or heterogeneous bioinks [13].

Despite advances in the field of 3D-bioprinting and the exploration of innovative bioinks, challenges remain, including the weak mechanical properties of natural biomaterials, inherent drawbacks of synthetic materials (e.g., poor biocompatibility, lack of bioactive ligands, and cytotoxicity), and the arduous task of bioprinting materials to mimic all structural, physical, and biological properties of the natural ECM [14]. One of the most notable obstacles to the successful implementation of 3D-bioprinting is the relatively narrow range of bioprintable and biocompatible materials available [14]. This review aims to bridge this gap by providing a critical overview of emerging nanomaterial fillers designed to create ideal heterogeneous and composite bioinks for 3D-bioprinting of complex functional tissues. First, we discuss the rational design of bioinks to achieve fully functional 3D tissues with complex geometries along with the criteria that need to be considered when choosing a bioink for bioprinting. We then discuss the role of fillers in the development of the next generation of bioinks, and we highlight the diverse range of stimuli-responsive nanofillers that respond to stimuli, such as magnetic fields, electrical fields, and light. We also discuss the emerging application of nanofillers as a contrast agent for real-time monitoring of implanted 3D-bioprinted scaffolds. Acquiring information through in vivo imaging and tracking methods is crucial for improving the design and bioprinting of scaffold systems toward the goal of personalized tissue engineering. Finally, we will cover concerns related to the application of nanomaterials as fillers and the future direction of 3D-bioprinting.

## Rational Design of Bioinks to Achieve Fully Functional 3D Tissues with Complex Geometries

Bioink properties are influenced by material choice and printing parameters. In some cases, different applications, cell types, and printing modalities may require a combination of different bioink formulations. To highlight the importance of bioinks in the construction of fully functional 3D tissues, it is helpful to understand the state of the knowledge in 3D-bioprinting technology. In their recent article, Ji and Guvendiren provide a comprehensive overview of the evolution of 3D-bioprinting technology and emerging approaches, such as novel omnidirectional bioprinting, volumetric bioprinting, microfluidic-assisted bioprinting, 4D-bioprinting, in situ bioprinting, and aspiration-assisted freeform bioprinting [3]. Valot et al. illustrate the importance of designing bioinks based on different bioprinting modalities and highlight that it is generally more cost-effective for the bioink to be adapted to the printer rather than modifying the printer hardware [15]. The five main bioprinting techniques as described by Valot et al. are as follows: (i) extrusion and co-axial extrusion; (ii) fused deposition modeling (FDM); (iii) inkjet printing; (iv) laser-assisted printing; and (v) stereolithography [15].

In extrusion bioprinting, the most widely used bioprinting modality, the bioink is pushed through a nozzle to form a ribbon that produces a base layer in a desired shape and the print is then completed in a layer-by-layer fashion. FDM is similar to extrusion printing, but heat is applied to alter the physical state of the material as it exits the printhead. While FDM is used widely for thermo-responsive polymers and plastics, its application in bioprinting remains limited due to the high temperatures required for fusing the material and its incompatibility with cells. Inkjet printing is a droplet-based method that involves the ejection of a small volume of bioink via a thermal, piezoelectric, electrostatic, or electro-hydrodynamic actuator. However, inkjet printing is not amenable to highly viscous bioinks. Laser-assisted bioprinting is a costly technique that involves a laser beam that penetrates a transparent slide coated with a layer of gold or titanium and a cell suspension to transfer droplets of the cell suspension onto a substrate. Stereolithography is characterized by the cross-linking of a bioink onto the surface of a vat of uncured liquid bioink containing cells. This technique provides high resolution but can be costly, requires a large amount of bioink, and can suffer from settling of suspended materials, such as cells or other particles during the printing process [15]. As extrusion is by far the most widely used bioprinting modality [15, 16], the remainder of this review will focus on bioinks used for extrusion bioprinting.

**Fig. 2:**
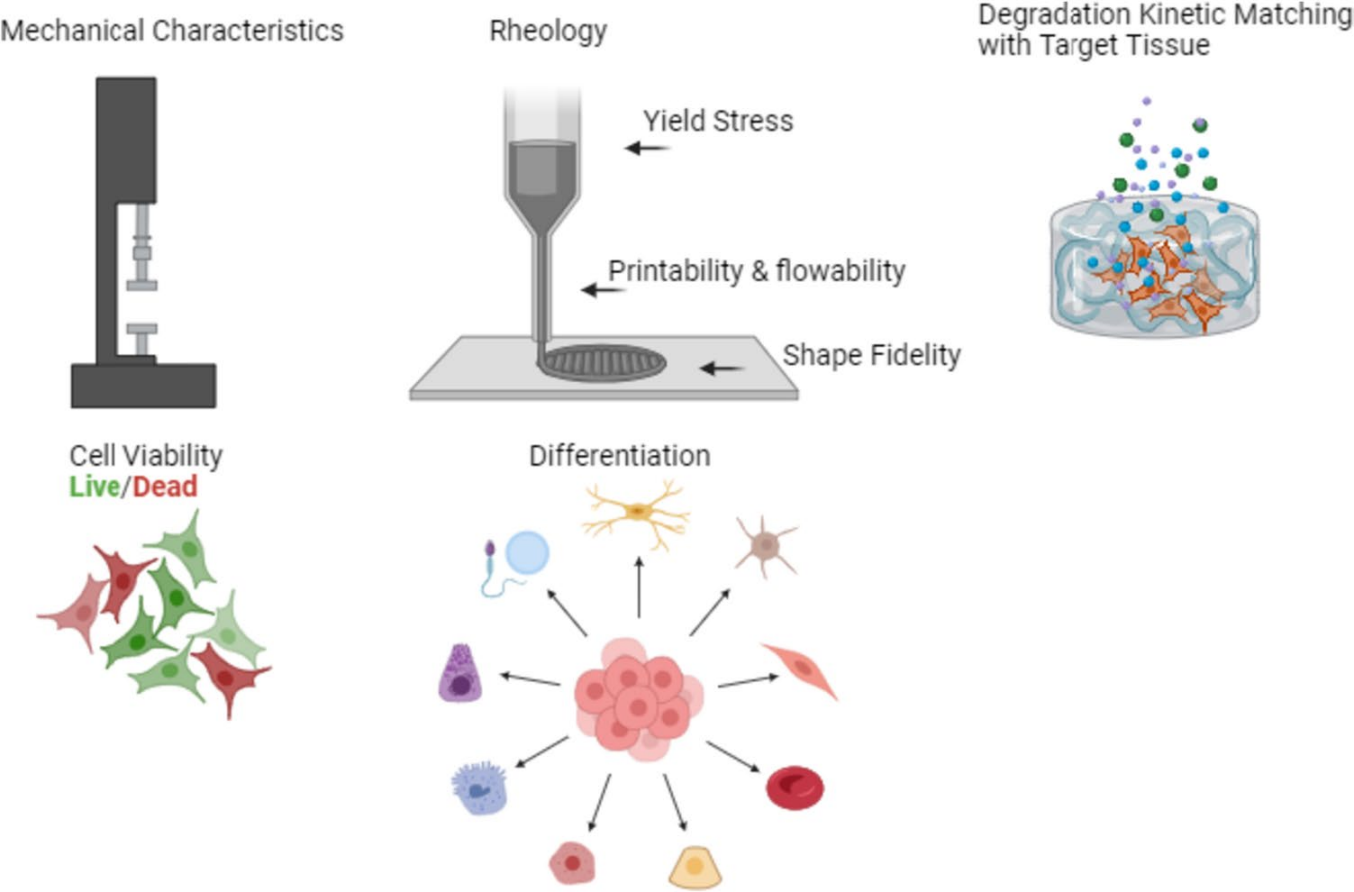
The key parameters for consideration in the design of an advanced bioink. Figure created using BioRender

One of the main advantages of extrusion-based bioprinting is its ability to deposit high cell densities that approach physiological cell densities [16]. At the pre-extrusion stage, viscosity, cell distribution, and biocompatibility are critical bioink properties, while at the mid- and post-extrusion stages, shear stress minimization, and physiological stability, respectively, are essential [17]. In any application, the ideal bioink material should demonstrate sufficient printability, high mechanical and structural integrity once crosslinked or gelled, stability, and a degradation matched with the regeneration of the target tissue. It should also be cytocompatible, non-immunogenic, and promote cell attachment [18]. Bioink properties that influence printability include shear thinning, recoverability, gelation kinetics, biocompatibility, and biodegradation [17]. Viscosity and shear thinning are critical to minimize applied stress on the cells and improve cell viability during the printing process, and degradability, cell-instructive matrix remodeling, and ECM production are key biochemical considerations of the final bioprinted structure [17]. Figure 2 summarizes the characteristics of ideal bioinks. Several factors that should be considered when choosing a bioink for bioprinting are discussed below.

### Shear Thinning

Shear thinning is an important property in bioinks used for 3D-bioprinting and is defined by a decrease in the viscosity of a polymer solution as the shear rate increases. Many polymeric materials that form hydrogel exhibit shear thinning when extruded through a nozzle, which is particularly beneficial as this decreased shear stress can be tolerated by cells. Moreover, after the bioink is extruded, the shear rate decreases significantly as viscosity increases, which allows the fluid to stay in place long enough for it to become crosslinked or thermally set into a more stable hydrogel construct [19]. Determining viscosities across a range of shear rates from low (< 10^–3^ s^−1^) to high (> 10^2^ s^−1^) is commonly used to predict the behavior of a bioink during the printing process, while other methods involve applying models to describe the ability of a hydrogel to shear thin [17]. A high viscosity at low shear rates and a low viscosity at high shear rates is usually desired for an extrusion process, and the rheological characteristics of the ink in the presence of a nanomaterial additive must always be considered when developing a heterogeneous bioink containing a nanofiller.

In terms of printing parameters, it is important to consider the conditions of the environment to which cells are exposed during the printing process, as they may not be conducive to cell survival. For example, during the bioprinting processes, cell damage may result from shear, thermal stress, and radiative stress, and the magnitude of cell damage is dependent on the strength and duration of the stressor [20]. To ensure the bioink matrix can protect the cells from damage during printing, bioink properties as well as technical parameters (e.g., nozzle size and geometry) must be considered [8]. To improve the viability of printed cells, adjusting the printing speed to reduce exposure time can be considered, although this may also increase the shear stresses that the cells experience. Alternatively, the printing environment can be optimized to simulate the conditions of an incubator, which can enhance cell viability during the printing process [21]. Typical nanomaterial additives do not have deleterious effects on cells during the printing process, but one must consider that many nanomaterials can be recognized, and in some cases, can be engulfed by cells, leading to potential alterations in cell behavior [22].

### Viscosity and Viscoelasticity Properties

During printing of the bioink strands, sufficient viscosity is crucial to maintain their cylindrical profile and ensure uniform cell and nanomaterial dispersion. The viscosity range of bioinks that can be utilized for extrusion bioprinting lies between 30 and 6 × 10^7^ mPa [23]. Prior to bioprinting, the bioink requires preconditioning with controlled holding time and temperature. The viscoelasticity of the bioink plays a vital role in ensuring adequate printability and shape retention to support the long-term mechanical demand of the structures. Notably, an increase in elasticity during extrusion elevates the shear stress on the cells, which can cause cell damage as discussed above. Thus, an ideal bioink must have an appropriate viscosity to prevent such damage during the bioprinting process [24]. However, highly viscous bioinks need high pressure for extrusion through the nozzle of the printer, which can result in high shear force and damage to cells. Natural polymers, which have high molecular weight, provide the necessary viscosity at low concentrations, resulting in adequate cell dispersion for subsequent cell proliferation and migration [25, 26]. While the optimal concentrations of polymer are cell and additive dependent, studies recommend concentrations less than 10% or ideally less than 5% [27].

### Printability

In bioprinting, printability refers to the ability of a bioink to be printed in the intended shape and maintain its structure. This is influenced by numerous factors, such as viscosity, surface tension, cross-linking mechanisms, and other rheological properties of the bioink [26]. Printability can be categorized by the morphology of the extruded samples as: under gelation, proper gelation, or over gelation. Proper gelation produces smooth surfaces with regular grid patterns, whereas under gelation allows the printed features to flow together in circular patterns rather than squares, and over gelation exhibits irregular grid patterns [28].

The characteristics of nanofillers, including their shape, surface area, and topography, has potential to affect the printability of the bioink. Despite the potential of this field, there are a paucity of research studies that explore the impact of nanomaterial characterization on printability. Bednarzig et al. evaluated the influence of different particle shapes of bioactive glass (angular and round), surface area, and surface topography on the printability of an alginate-based bioink and demonstrated that filler surface area had the greatest impact on printability. The study also showed that with a higher effective surface area of particles, better printing results were achieved without the need for a higher filler content. Moreover, changing the particle shape could alter the critical filler concentration required for printability. The comparison of bioinks with smooth and round glassy filler particles to those with angular particles demonstrated that the latter had a more substantial impact on the rheological properties of the bioink. Composites with angular particle morphologies showed high resolution during the printing process, while composites with comparable amounts of round filler particles lacked stackability after printing [29].

### Cross-linking and Gelation

To achieve high printability, a highly viscous cell-laden polymer-based bioink is generally recommended. However, high viscosity of the bioink can have a detrimental effect on cell viability. To mitigate this issue, a bioink with sufficient viscosity that can rapidly crosslink post-printing may be employed to achieve superior fidelity of the printed structure. Indeed, hydrogels with higher moduli obtained through post-printing cross-linking may be necessary for surgical implantation and handling. Additionally, such hydrogels can provide appropriate stiffness to the cells, resulting in longer degradation rates both in vitro and in vivo. The cross-linking process is a crucial aspect of the development of the final printed scaffold structure, as it has a direct impact on the resolution of the printed structure. The process should be fast, and the bioink must possess tissue-matching mechanics afterward without having toxic effects on cells.

There are two main types of cross-linking processes used for bioprinting of hydrogels: chemical and physical cross-linking. Chemical cross-linking occurs when covalent bonds are formed between polymeric chains, usually through the addition of chemical crosslinkers, such as sodium bicarbonate (for cross-linking individual collagen layers) [30], calcium chloride for alginate [31], or sodium tetraborate (for polyvinyl alcohol (PVA)) [32], or through various chemical reactions, including Schiff base chemistry, azide-alkyne cycloaddition, hydrazide-aldehyde coupling, thiolene coupling, enzymatic cross-linking, or light exposure (e.g., ultraviolet (UV), visible, or near-infrared light) [33]. While chemically cross-linking hydrogels can improve shape stability, the cross-linking kinetics should be precisely controlled to prevent printer nozzle blockage. In contrast, the physical cross-linking process occurs through the formation of noncovalent bonds, such as H-bonds, hydrophobic interactions, electrostatic attraction, and ionic cross-linking. These hydrogels are generally mechanically weaker but offer a more cell-friendly environment than chemically crosslinked hydrogels. To further improve the stability of 3D-bioprinted constructs and potentially aid in the cross-linking process, nanofillers or chemical functionalities can be introduced [33, 34]. Besides cell viability, the total degree of cross-linking, which includes both primary and secondary cross-linking, can impact cell spreading and the formation of cellular networks. Research has shown that when using cast hydrogels, increasing the degree of cross-linking or polymer fraction can negatively affect cell viability and the formation of cellular projections and networks [35–37].

### Cell Viability and Biocompatibility

As alluded to above, a considerable challenge in 3D-bioprinting is maintaining cell viability, which impacts subsequent cellular events, such as proliferation, differentiation, and tissue integration. One approach to enhance cell survival and proliferation is the use of natural bioink materials with cell adhesive sites and sufficient hydrophilicity. Such natural bioinks may withstand harsh fabrication conditions but suffer from batch-to-batch variability [38]. Synthetic polymers, on the other hand, overcome this variability but do not offer natural cell adhesive sites [39]. The biocompatibility of all materials used in bioinks is essential to achieving high cell viability. The biomaterials must demonstrate low cytotoxicity, be non-immunogenic, and not release toxic by-products during the degradation process. Additionally, the bioink and bioprinting process must be biocompatible to minimize stress on the cells as much as possible as discussed above [40].

In general, cells tend to favor porous structures with cell adhesive properties, which helps promote cell spreading and migration. The structure should also have proteolytic cleavage sites that allow for remodeling by cells. While increasing the polymer concentration and crosslink density can lead to improved print fidelity, this approach can also reduce the porosity of the structure, which limits the available space for cells to spread and migrate. With low porosity, nutrient diffusion can also become limited [41, 42]. In contrast, it is possible to use nano- or micro-fillers with rapid degradation kinetics as porogens, as demonstrated by Rodenas-Rochina et al., using a highly porous matrix composed of poly (l-lactide)/poly (ε-caprolactone) (PLLA/PCL) and a HAp nanofiller as promising materials for healing large bone defects [43].

## Nanomaterials as Fillers for 3D-Bioprinting and Engineering of Tissues

**Fig. 3:**
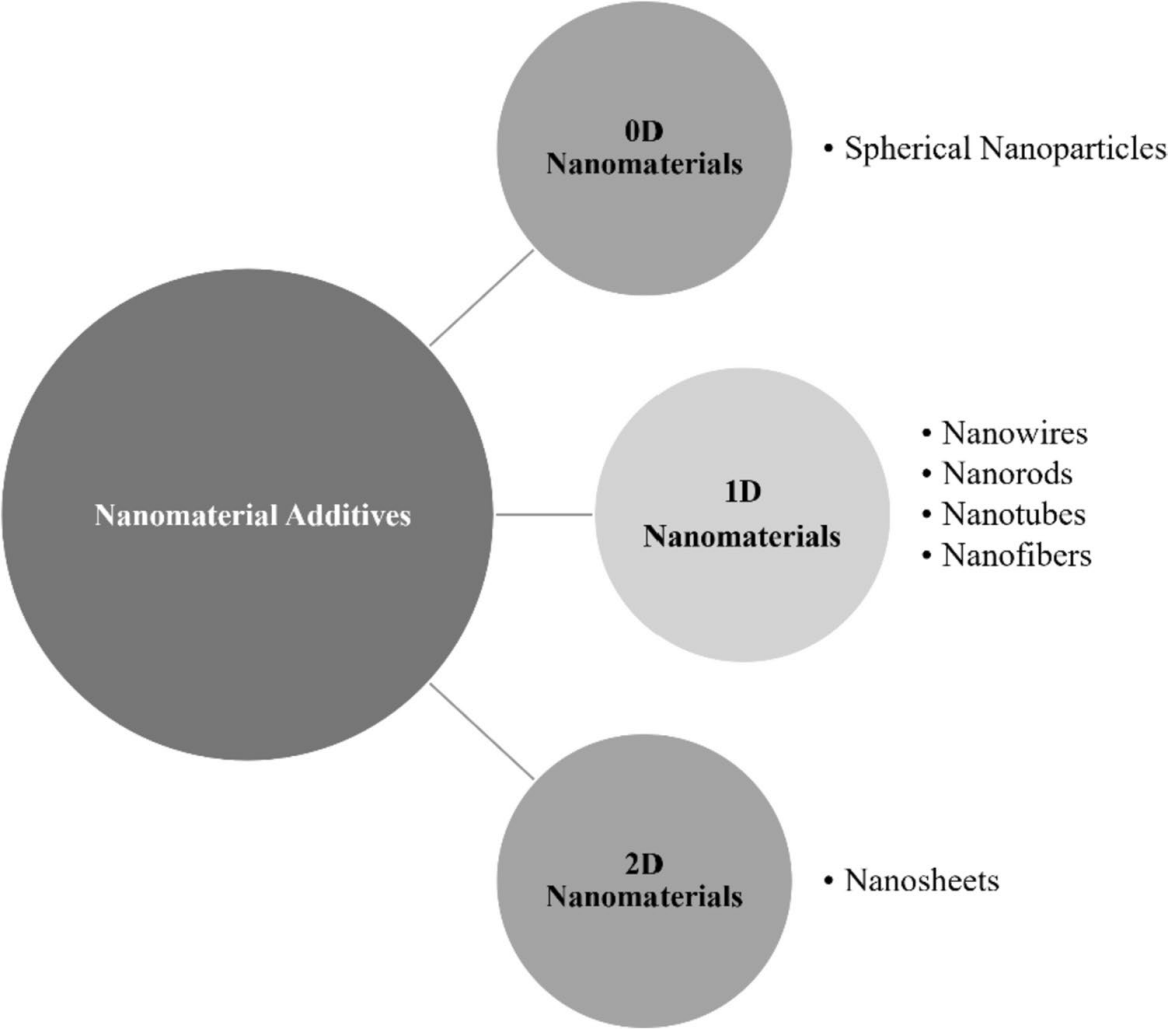
Different classes of nanomaterials as fillers in bioinks for 3D-bioprinting

Nanomaterials have been studied for different biomedical applications, such as drug delivery systems and controlled drug release, tissue-engineered scaffolds, dressings for wound healing, biosensors, biomedical devices, and cosmetics [44–47]. As described in general terms above, incorporation of nanomaterials into bioink can significantly improve the physiochemical properties of the bioink (e.g., increased shear thinning) and enhance shape fidelity, stiffness, and degradation kinetics of the bioprinted structure when exposed to physiological conditions [48, 49]. Such nanomaterials can be classified into three groups based on their dimensional properties: 0D, 1D, and 2D (Fig. 3).

Zero-dimensional (0D) nanomaterials are characterized by having all three dimensions (*x*, *y*, and *z*) at the nanoscale (> 10 nm) [50] and include metallic and metallic oxide spherical nanoparticles, such as silver and gold nanospheres, iron oxide, and zinc oxide nanoparticles, and non-metallic materials, such as silica nanoparticles, calcium phosphate nanoparticles, nano-hydroxyapatite (nHAp), graphene oxide nanoparticles, bioactive glass nanoparticles, and polypyrrole spherical nanoparticles, among many other examples. For example, Bednarzig et al. investigated in vitro biocompatibility with bioactive glass particles of different shapes (angular and round) for bone regeneration and found that the 3D-bioprinted structure formed an interconnected porous scaffold and showed increased cell viability and proliferation in the presence of the bioactive glass [29]. One-dimensional (1D) nanomaterials are characterized by having two of their three dimensions (x, y) in the nanoscale range with the third dimension of the nanostructure outside the nanoscale range (> 10 nm). Examples of 1D nanomaterials include silver nanowires, gold nanorods, carbon nanotubes (CNTs), carbon nanofibers (CNFs), collagen nanofibers, and cellulose nanofibrils/nanofibers (CNFs). Navaei et al. fabricated UV-crosslinkable gold nanorod-incorporated gelatin methacrylate (GelMA) hybrid hydrogels to promote electrical conductivity and mechanical stiffness of the hydrogel matrix and found that cardiomyocytes seeded on GelMA-GNR hybrid hydrogels exhibited high viability and metabolic activity [51]. Finally, 2D-dimensional (2D) nanomaterials are described as having two dimensions outside the nanometer range and one dimension (*x*) at the nanoscale (ranging from 1 to 100 nm). Examples of 2D nanomaterials include disk-like Laponite™ nanosilicates (nSi), graphene, and MXene nanosheets. For example, Ahlfeld et al. developed a bioink using Laponite™ (a synthetic nanosilicate clay) blended with alginate and methylcellulose and demonstrated that, following extrusion, 70–75% of printed immortalized human mesenchymal stem cells survived and that cell viability was maintained over 21 days [52]. Table 1 provides an overview of recent studies related to the application of various classes of nanomaterials as fillers for 3D-bioprinting.

**Table 1:** Overview of recent studies related to the biomedical application of various classes of nanomaterials as fillers for 3D-bioprinting

	Filler/additive	Ink material(s)	Cell line(s)/type(s)	References
Zero-Dimensional (0D)	Bioactive glass NPs	Alginate/gelatin	SaOS-2, hBMSC, mBMSC	[53, 54]
		Alginate dialdehyde/gelatin	MG-63, rMSC	[55, 56]
	Calcium phosphate	Alginate/polyvinyl alcohol	MC3T3-E1	[57]
	Carbon dot NPs	Collagen	BMSC	[58]
	Hydroxyapatite	Alginate	BM-MSC	[59]
		Alginate/gelatin	hMSC, hASC, hAdSC, mouse chondrocyte	[60–64]
		Alginate/polyvinyl alcohol	MC3T3-E1	[65]
		Chitosan/alginate	MC3T3-E1, hASC	[65, 66]
		GelMA	hASC	[67]
		GelMA/HAMA (hyaluronic acid methacrylate)	hASC, AdMSC, femoral condyle chondrocyte	[62, 66, 68]
		HAMA/gelatin	AdMSC	[69]
	Iron oxide	Agarose/collagen	Human knee articular chondrocyte	[70]
		Collagen	hiPSC-CM	[71]
	Keratin-coated gold NPs	GelMA/alginate	U87-MG	[72]
	Poly-3,4-ethylene dioxythiophene NPs	GelMA	C2C12	[73]
	Polypyrrole spherical NPs	Type I collagen	PC12	[74]
		Type I collagen	PC12	[75]
One-Dimensional (1D)	Cellulose nanofibrils/nanofibers	Alginate	Human nasal chondrocyte	[76]
	Carbon nanotubes	Carboxymethylated cellulose nanofibrils	SH-SY5Y	[77]
		Collagen I	P2 rat DRG	[78]
		Collagen methacrylate/alginate	HCAEC	[79]
		GelMA	Neonatal rat ventricular cardiomyocyte	[80]
	Gold nanorod	GelMA	Cardiac fibroblast/cardiomyocyte	[81]
	Gold nanowires	Collagen	C2C12	[82]
	Hydroxyapatite nanorods	Alginate/chitosan	hASC	[65]
	Oxidized cellulose nanofibers	Alginate	Human meniscus fibrochondrocyte	[83]
	Polylactic acid nanofibers	Alginate	hASC	[84]
	Silk fibroin nanofibers	Chitosan	10T1/2	[85]
	Silver nanowire	GelMA/collagen	HUVEC	[86]
Two-Dimensional (2D)	Graphene oxide	Alginate	MSC	[87]
		Chitosan methacrylate	L929	[88]
		Collagen/chitosan	Human chondrocyte	[89]
		GelMA	Mouse neural stem cell, PC12, NIH-3T3, MC3T3-E1	[90, 91]
		GelMA/PEGDA	hBMSC	[92]
		Polyurethane dispersion	Neural stem cell	[93]
	Graphene oxide (reduced)	Decellularized porcine myocardial ECM	hiPSC-derived cardiomyocyte	[94]
	Laponite	Alginate methylcellulose	hTERT MSC	[52]
		PEGDA	Rat osteoblast	[95]
	MXene	GelMA/HAMA	hMSC	[96]
		Hyaluronic acid/alginate	HEK-293	[97]

Conventional bioinks for 3D-bioprinting do not always elicit desired biological responses, mainly due to the static nature of the 3D-bioprinted structure once printed, which contrasts with the dynamic and constantly changing morphologies of native tissues in response to external stimuli [98, 99]. It is therefore essential to develop innovative stimuli-responsive bioinks for bioprinting scaffolds that can undergo an intended transformation as required for specific tissues. Nanomaterials respond to stimuli such as magnetic fields, electrical fields, and light [58, 71, 78–82, 100, 101]. While stimuli-responsive nanomaterials are already a popular choice as fillers in hydrogel scaffolds produced by casting, their application in 3D-bioprinting remains relatively limited. Table 2 provides an overview of the numerous biomedical applications of stimuli-responsive materials, including their use as fillers for various tissue engineering applications.

**Table 2:** Overview of the numerous biomedical applications of stimuli-responsive materials used as fillers for 3D-bioprinting

Stimulus	Nanomaterial	Hydrogel	Cell type(s)	Application	References
Light	Gold nanorods	PNIPAAm/PEGDA	Smooth muscle cell, endothelial cell	Remodeling arteries	[102]
	Carbon dot NPs	Collagen	BMSC	Enhancing chondrogenesis	[58]
Magnetic	Iron oxide NPs	Collagen	hiPSC-derived cardiomyocyte	Cardiac constructs	[71]
Electric	Carbon nanotubes	Methacrylated collagen/alginate	HCAEC	Cardiac patch	[79]
		GelMA	Neonatal rat cardiomyocyte	Cardiac constructs	[80, 101]
			129/SVE-derived mouse stem cell	Cardiac differentiation of embryoid bodies	[101]
		Collagen	P2 rat DRG	Promoting neurite growth	[78]
	Gold nanorods	GelMA	Cardiac fibroblast, cardiomyocyte	Cardiac constructs	[81]
	Polydopamine-modified black phosphorus nanosheets	GelMA	MSC	Electroactive tissues	[103]
	Graphene	Decellularized ECM	hiPSCs	Heart tissue engineering	[94]
		Chitosan methacrylate	L929	Potential for tissue engineering	[88]
	Silver nanowires	GelMA/collagen	HUVEC	Potential for tissue engineering	[86]
	Gold nanowires	Collagen	Myoblast	Muscle regeneration	[82]

### Applications of Magnetic Nanofillers in 3D-Bioprinting

**Fig. 4:**
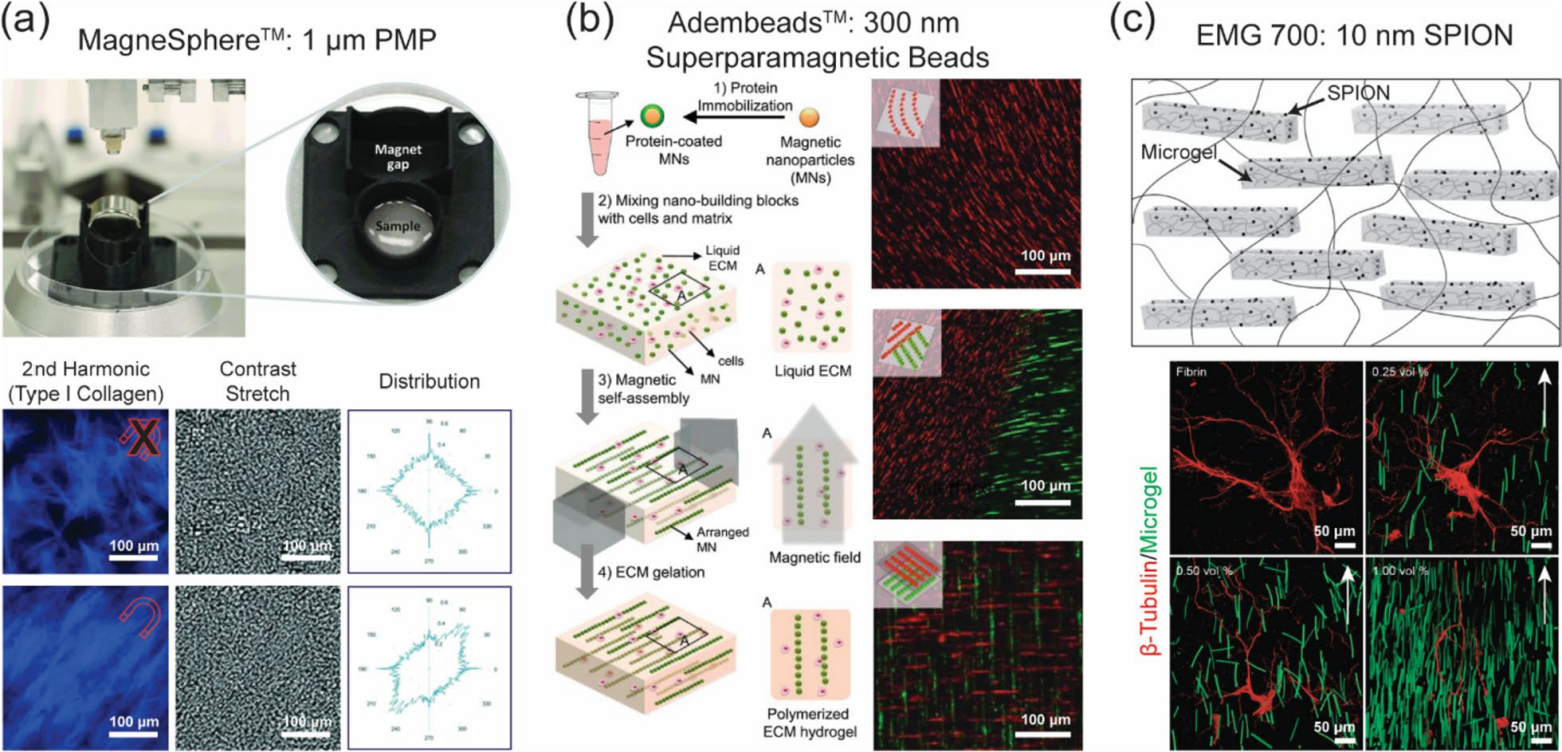
Magnetically responsive nanomaterials used in 3D-bioprinting. **a** A bioprinting stage with a custom-designed adaptor that incorporates a cylindrical magnet to force real-time remodeling of the bioink during bioprinting was used to promote collagen fiber alignment in a printable [70]; **b** Functionalized magnetic particles can be used to create diverse types of alignment in 3D in the presence of a magnetic field [104]; **c** Preparation of hybrid hydrogel (anisogel), which generates a unidirectional structure in situ by aligning magnetoceptive microgels [105]

The use of magnetic nanomaterials, most commonly iron oxide nanoparticles, has demonstrated significant potential in aligning ECM fibers due to their high sensitivity to externally applied magnetic fields. Betsch et al. achieved real-time magnetically directed collagen fiber alignment for the generation of complex multilayered tissues (with a specific focus on cartilage tissue engineering) by reinforcing iron nanoparticles in collagen-agarose ink to produce a magnetic-responsive construct (Fig. 4a). The study demonstrated that hydrogel blends with unidirectionally aligned collagen fibers exhibited significantly higher compression moduli compared to those with random fibers, which could be of great benefit to the field of tissue engineering of load-bearing and force-generating tissues. Bioprinted constructs with alternating layers of aligned and random fibers were fabricated, and after 21 days of cell culture, significantly higher collagen expression was noted compared to randomly oriented fiber constructs. The incorporation of a magnetic field during printing to achieve unidirectional fiber alignment in hydrogels resulted in a significant increase in compression moduli at up to 20% compressive strain.

To investigate the role of topographical cues on cell behavior, Jiyun et al. developed a tissue-mimetic platform that programs topographies in 3D biomaterials using magnetic field-directed self-assembly of surface-functionalized magnetic particles serving as the tissue building blocks (Fig. 4b). In this approach, ECM proteins were crosslinked to the activated surfaces of 300-nm superparamagnetic particles, which were then mixed with cells in liquid ECM or mixed in liquid ECM alone. A magnetic field was then applied to this mixture well before polymerization of the hydrogel. The applied magnetic field was sufficient to overcome the rheological resistance within the hydrogel, resulting in self-organization into specific geometric patterns with defined anisotropy achievable by simply applying and controlling the external field arrangement [104].

Rose et al. demonstrated a new type of anisotropic and hybrid hydrogel, called an anisogel [105]. An anisogel is composed of microgels doped with small quantities of superparamagnetic iron oxide nanoparticles (0.0046 vol %), allowing alignment in the presence of a low external magnetic field in the milliTesla range (Fig. 4c). Following alignment, the oriented microgels became interlocked within a crosslinked hydrogel. The resulting anisogel was used to investigate the sensitivity of fibroblasts and neurons to structural guidance cues in 3D and the minimal number of signals required to prompt initiation to grow in an aligned manner. The unidirectional orientation was strongly sensed by the cells and resulted in parallel neurite extension [105].

### Applications of Electrical Stimuli-Responsive Nanofillers in 3D-Bioprinting

**Fig. 5:**
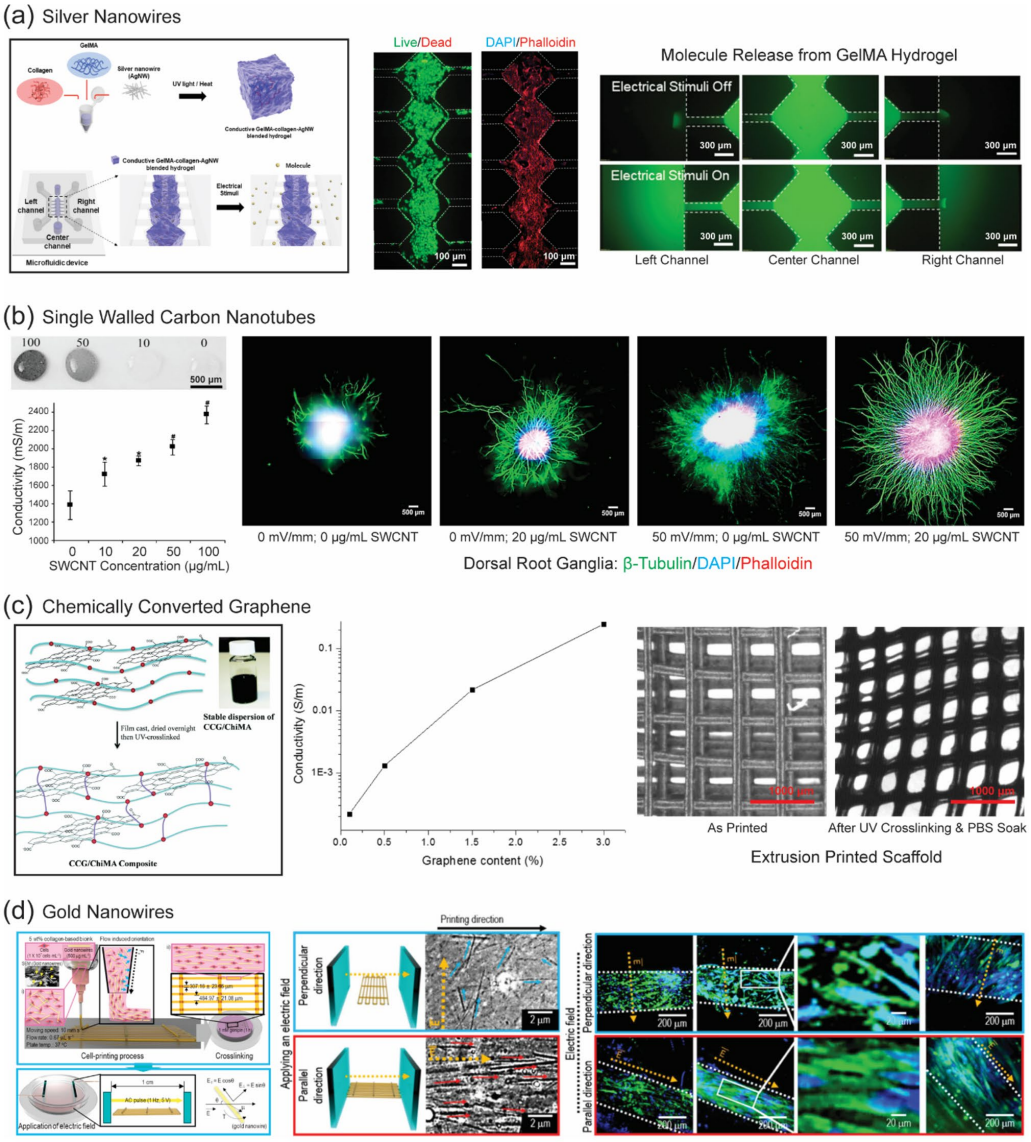
Use of electrically conductive nanofillers for 3D-bioprinting applications. **a** Strategy for generating conductive GelMA–collagen–AgNW scaffolds [86]; **b** Neurite outgrowth can be improved by either using electrical stimulation of 50 mV mm^−1^ or reinforcing the scaffold with 20-μg mL^−1^ SWCNT. At even low concentrations of SWCNTs, the electrical conductivity of collagen hydrogels can be significantly improved [78]; **c** CCG/ChiMA electrically conductive composite and representative bioprinting results [88]; **d** Extrusion bioprinting in conjunction with application of an electric field, results in the alignment of gold nanowires and surrounding microstructures [48]

Certain types of human tissues, such as neural, cardiac, and skeletal muscle tissue, respond to and generate electrical signals. These tissue types are also known to have limited regenerative potential. For example, is estimated that only 1% of cardiomyocytes undergo replacement annually at the age of 25, with this number dropping to 0.45% at the age of 75 [106, 107]. Electrical stimulation plays a significant role in the migration, proliferation, and guiding differentiation of the cell types that reside in these tissues [108–110]. Both the conductivity and mechanical properties of the construct are key factors in the selection of electroconductive filler for 3D-bioprinting of these target tissues (Fig. 5).

Another important advantage of incorporating electrically conductive nanomaterials into bioinks is facilitating the alignment of electrosensitive cell types, including cardiomyocytes, myoblasts, and neural stem cells, within the scaffold or on its surface. While electrosensitive cell types align parallel to the electric field vector due to the electrical stimulation causing rearrangement of the cell cytoskeleton, other types of cells (e.g., vascular endothelial cells, cardiac adipose tissue-derived progenitor cells, endothelial progenitor cells, bone mesenchymal stem cells, and adipose-derived stromal cells) align perpendicularly to the direction of the electric field to minimize the field gradient across the cell body. However, it has been observed that the effectiveness of electrically induced cellular alignment is not optimal and requires a prolonged stimulation period, which may ultimately adversely impact cell viability [111]. To address this, electroconductive nanomaterials such as carbon nanotubes, graphene nanosheet, gold nanowires, and electroconductive polymers (e.g., of ethylenedioxythiophene and pyrrole) have been added into bioinks to guide the alignment of electrically sensitive cells. The premise is that electroconductive nanomaterials can align themselves with the direction of an applied electric field, thereby assisting in the alignment of the surrounding microstructures.

In selecting metallic nanoparticles as a conductive filler to reinforce the bioink, it is essential to consider factors, such as electrical conductivity, oxidation, cost, and desired electrical and magnetic properties. These factors depend on the specific requirements of the printed scaffold. It is essential that 3D-printed conductive scaffolds must have minimal electrical resistance to reduce heating effects. Therefore, materials with superior electrical conductivity, such as silver, gold, and copper, are preferred for use in conductive metallic nanoparticle inks. Some metallic nanoparticles tend to form metal oxides when exposed to air, making them unsuitable for formulating conductive bioinks. For example, copper nanoparticles rapidly oxidize in the atmosphere, and the resulting copper oxides (CuOs) are significantly less conductive than pure copper nanoparticles [112]. The cost of bioink materials is also important to consider, especially in cases where mass production of the scaffolds is required. For example, gold nanoparticle bioinks have excellent electrical properties and oxidation stability but potentially introduce higher costs for the scale-up and use in most applications than other types of nanoparticles. Therefore, ongoing research is being conducted to find suitable materials for formulating metallic nanoparticle fillers that possess good electrical and material properties at a significantly lower cost.

Materials with desired electrical properties for specific tissue scaffolds may also be considered for the choice of ideal conductive nanoparticles [113]. Ha et al. created a conductive gelatin methacrylate (GelMA)-collagen scaffold with the reinforcement of silver nanowires (AgNWs) in a bioink containing human umbilical vein endothelial cells (HUVECs) [86]. The storage modulus and loss modulus of the GelMA-collagen hydrogel were significantly improved to 13.77 and 201.8 kPa, respectively, compared to collagen alone. Moreover, the storage modulus increased from 2.2 to 13.77 kPa with the addition of AgNWs, indicating that AgNWs played a crucial role in improving the electrical conductivity and mechanical strength of the blended hydrogel. Finally, the 3D-bioprinted scaffold demonstrated the formation of a vascular network of HUVECs (Fig. 5a) [86].

In another study, single-walled carbon nanotubes (SWCNTs) were used as a filler to create an electrically conductive 3D scaffold [78]. SWCNT loadings ranging from 10 to 100 μg mL^−1^ were examined and enhanced neurite outgrowth was observed at a loading concentration of 20 μg mL^−1^ relative to the filler-free scaffold. Furthermore, when exogenous electrical stimulation (50 mV mm^−1^) was applied, dorsal root ganglia (DRG) neurite outgrowth was greater, resulting in a 7.0-fold increase in neurite outgrowth compared to the filler-free control scaffold. This study concluded that increases in neurite outgrowth do not appear to be influenced by mechanical properties, as the elastic modulus did not significantly change despite an increase in conductivity (Fig. 5b) [78]. These findings suggest a promising avenue in neural tissue engineering. The ability to modulate neurite outgrowth through precise control of nanomaterial loading and electrical stimulation provides valuable insights for designing bioinks with tailored properties for neural tissue engineering applications.

Ahadian et al. designed a dielectrophoretically aligned CNT GelMA hydrogel with tunable mechanical and electrical characteristics, which enhanced the cardiac differentiation of mouse embryoid bodies (EBs) compared with pure GelMA and GelMA containing randomly oriented CNTs. The resulting constructs expressed key cardiac genes, such as Tnnt2, Nkx2-5, and Actc1, were positive by troponin-T immunostaining and exhibited beating [101]. Recent research has suggested that incorporating graphenic materials such as graphene oxide (GO) and graphene can significantly enhance the properties of such constructs [114]. Graphene, which is a monolayer of carbon atoms arranged in a honeycomb lattice, possesses exceptional mechanical, electrical, optical, thermal, and magnetic properties, thus exhibiting advantages over other commonly used carbon structures.

Tsui et al. created a hybrid electroconductive hydrogel scaffold composed of decellularized porcine myocardial extracellular matrix (dECM) and reduced graphene oxide (rGO) [94]. The process of decellularization preserved the tissue-specific protein profile. By modulating the rGO content and degree of reduction, they were able to tune the mechanical and electrical properties of the hydrogels. Using the dECM-rGO hydrogel scaffolds and human-induced pluripotent stem cells (hiPSCs)-derived cardiomyocytes, they generated engineered heart tissues (EHTs) that exhibited increased twitch forces and higher expression of genes that regulate contractile function. Furthermore, these EHTs exhibited improvements in electrophysiological function, including action potential duration, calcium handling, and conduction velocity by the reinforcement of rGO as an electroconductive filler [94]. The development of hydrogel-based cardiac implants that are electrically conductive, mechanically robust, and biologically functional represents an area of intensive research. Despite considerable progress, achieving all three of these critical characteristics remains a challenge. Researchers continue to investigate various approaches to optimize the performance of hydrogel-based cardiac implants, with the aim of creating a scaffold that can effectively integrate with the surrounding tissue and support cardiac function over the long term. Alginate [115], collagen [116], gelatin [117], fibrin [118], and poly(N-isopropyl acrylamide) (PNIPAAM) [119] are some of the commonly used hydrogels in cardiac tissue engineering with potential to be applied in 3D-bioprinting.

Sayyar et al. developed a conductive 3D scaffold from methacrylated chitosan (ChiMA) containing graphene nanosheets [88] (Fig. 5c). Reinforcement with graphene improved the mechanical and electrical properties of the scaffold, as well as the adhesion, proliferation, and spreading of L929 fibroblast cells cultured in the scaffold. The conductivity of the scaffold increased as the chemically converted graphene (CCG) content increased. The conductivity of the ChiMA 0.1CCG composite was ~ 2E-4 S m^−1^, which increased to ~ 1.5E-3, 2E-2, and 1.33 E-1 S m^−1^ upon increasing the CCG content to 0.5, 1.5, and 3 wt %, respectively [88].

Another study featured a conductive hydrogel scaffold based on the integration of polydopamine-modified black phosphorous (BP@PDA) nanosheets into GelMA hydrogels [103]. Adding the electroconductive filler significantly enhanced the electrical conductivity of the hydrogels and improved cell migration of mesenchymal stem cells (MSCs) within the 3D scaffold. According to gene expression and protein-level assessments, the incorporated BP@PDA significantly promoted the differentiation of MSCs into neural-like cells under synergistic electrical stimulation [103]. Similarly, Zhu et al. bioprinted a conductive cardiac tissue scaffold by incorporating gold nanorods (GNRs) into a GelMA-based bioink [81]. In the scaffolds, cardiac cells showed improved cell adhesion and organization as well as enhanced rhythmic contraction compared to a filler-free scaffold [81].

As skeletal muscles are composed of elongated electrically responsive fibrous bundles of multinucleated myotubes that originate from the fusion and differentiation of satellite cells, muscle tissue represents another important target for development of conductive scaffolds. To ensure successful regeneration of muscle tissue, it is imperative to provide structural guidance to the muscle cells, thereby inducing the effective differentiation of skeletal muscle cells [120, 121]. To achieve this goal, as well as to accelerate the alignment of the cells and mimic the electrical properties of muscle tissue, Kim et al. fabricated a myoblasts-laden collagen-based bioink for the 3D-bioprinting of muscle using gold nanowires (GNWs) as an electric stimuli-responsive filler (Fig. 5d). GNWs reinforced in a myoblast-laden collagen bioink significantly narrowed the fiber orientation distribution, favoring myogenic differentiation compared to electrically stimulated myoblast-laden collagen constructs in the absence of GNWs and non-electrically stimulated constructs. Furthermore, after implantation, muscle tissue regeneration was observed [82].

### Applications of Photo-Responsive Nanofiller in 3D-Bioprinting

Light-sensitive hydrogels can be classified as either UV- or visible light sensitive. Another class of nanoparticles called metal nanoshells, which consist of a dielectric core nanoparticle surrounded by an ultrathin metal shell, is emerging [122]. These nanoshells are capable of absorbing near-infrared (NIR) light wavelengths between 800 and 1200 nm, which can be transmitted through tissue with little attenuation due to the low absorption coefficients of water and hemoglobin on either side of this wavelength window [123]. This NIR absorption characteristic has enabled the development of a new class of light-sensitive hydrogels containing optically active metal nanoshells that can undergo a reversible volume phase transition in a temperature-sensitive polymeric matrix when irradiated with a laser, converting light energy to heat energy [124].

Generally, stimuli-responsive nanomaterials, especially light stimuli-responsive nanomaterials, are already widely used in non-printed 3D hydrogel scaffolds. Conversely, their applications in 3D-bioprinting are still extremely limited. To create a light-responsive hydrogel, researchers have incorporated nanomaterials, such as gold nanorods, carbon dot nanoparticles (CD NPs), gold–silver nanocores, gold–gold nanoshells, and SiO_2_-gold nanoshells into temperature-responsive interpenetrating polymer networks. By absorbing light irradiation, the nanoparticles transfer heat to the polymer networks, resulting in a change in the printed structure [58, 100, 123–125]

**Fig. 6:**
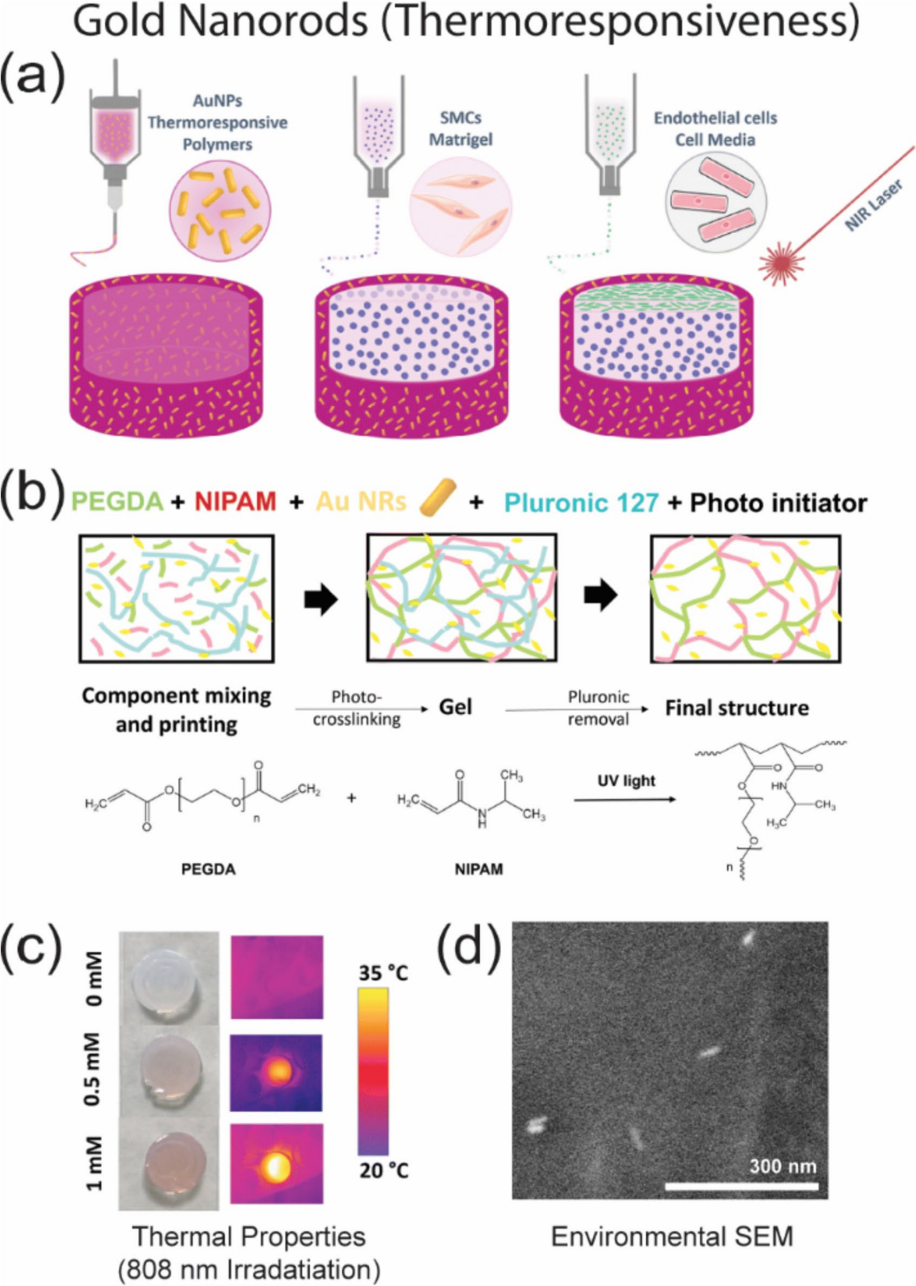
An example of 3D-printed contractile artery wall involving a three-step process **a** using a light-sensitive (thermoresponsive) bioink formulation **b** that can be considered both composite and heterogeneous [102]. **c** The maximum temperature increased as a function of AuNR concentrations using different laser powers under 808-nm laser irradiation. **d** Environmental scanning electron microscopy (ESEM) imaging of AuNRs in thermo-responsive gel

Lu et al. designed injectable hybrid collagen hydrogels to enhance chondrogenesis by photodynamic therapy (PDT), where collagen was crosslinked with CD NPs using genipin as a linker (termed collagen-genipin-CD NPs (CGN)) [58]. The CGN hydrogel showed increased stiffness due to the cross-linking effect of genipin and the presence of CD NPs and could produce a moderate amount of reactive oxygen species (ROS) through PDT. A regulated basal level of ROS is essential and advantageous to cell growth and differentiation. The hydrogel seeded with bone marrow-derived MSCs (BMSCs) and the subsequent PDT treatment were found to synergistically promote the in vitro chondrogenic differentiation and ectopic/orthotopic cartilage regeneration. The CGN hydrogel presented a 21-fold higher compression modulus and a 39.3% lower degradation rate than the pure collagen hydrogel. A combination of both PDT and CGN hydrogel increased the BMSCs proliferation by 50.3%, upregulated their expression of cartilage-specific genes, and enhanced glycosaminoglycan (GAG) secretion by 205.1% on day 21. This combination also accelerated the cartilage regeneration within 8 weeks. The stiffness enhancement and ROS generation synergistically contributed to chondrogenic differentiation by regulating the TGF-β/SMAD and mTOR signaling pathways, respectively. The CGN hydrogel displayed a remarkably higher compression modulus and a considerably lower degradation rate than the pure collagen hydrogel. This stimuli-response material thus represents a promising potential therapeutic option for cartilage regeneration and repair [58]. In another study to design a 3D contractile artery wall model, gold nanorod (AuNRs) as a photo-responsive plasmonic filler were incorporated into a thermo-responsive bioink composed of poly(N-isopropylacrylamide (thermo-responsive polymer), polyethylene glycol diacrylate (photo-curable polymer), and Pluronic 127 (as a sacrificial porogen material), resulting in changes in the thermo-responsive hydration state and volume in an on–off manner when stimulated [102]. The transition temperature and pulsatility were effectively tuned by adjusting the concentration of AuNRs (Fig. 6) [102].

### Use of Nanofillers for Real-Time Monitoring of Implanted 3D-Bioprinted Scaffolds

The field of tissue engineering and regenerative medicine has seen remarkable progress with the aid of imaging techniques. In vivo imaging and tracking methods are invaluable in providing critical insights into various aspects of engineered tissue constructs post-implantation, including the 3D geometrical structure of the scaffold, the interaction between biological molecules and cells with the scaffold, and the viability of cells within the constructs. Acquiring information through in vivo imaging and tracking methods is crucial for improving the design and bioprinting of scaffold systems to approach the goal of personalized tissue engineering.

Recent developments in the use of advanced bioinks for 3D-bioprinting, which incorporates nanomaterials as a contrast agent, have enabled the detection and tracking of cell populations within 3D-bioprinted scaffolds. This technology also allows for the real-time tracking of scaffolds both in vitro and after implantation in vivo. For instance, iron oxide nanoparticles, used as a magnetic resonance imaging (MRI) contrast agent, and gold nanoparticles, used as a computed tomography (CT) and fluorescence spectroscopy contrast agent, have been applied for real-time monitoring of the implanted scaffold positioning, integrity, and degradation [126, 127]. A recent study featured the use of gold nanoparticles as contrast agents, which were covalently conjugated to collagen. This resulted in the fabrication of a CT-visible collagen scaffold, and the X-ray attenuation of the conjugated scaffolds was used to measure hydrogel degradation over time in vitro [126]. In another study, iron oxide nanoparticles were employed as an MRI contrast agent to label and monitor collagen-based cardiac patches post-implantation onto the epicardial surface of the mouse heart. The T2*-weighted magnetic resonance images effectively highlighted the strong capability of this strategy in visualizing the engineered patch scaffold in a non-invasive manner [127]. Finally, incorporation of superparamagnetic iron oxide nanoparticles (SPIONs) into GelMA-based bioinks can provide the dual function of contrast in MRI and antibacterial activity against *Staphylococcus aureus*, as examined in a simulated in vitro model of a bacterial-contaminated scaffold [128].

## Conclusion

Nanomaterials hold significant promise in various fields, especially as fillers for 3D-bioprinting. It has been observed that incorporating nanomaterials as fillers in bioinks can lead to several favorable properties, such as improved electrical conductivity (by reinforcing electroconductive nanomaterials like CNTs, GOs, and Gold NPs), better mechanical properties (including increased shear thinning and improved shape fidelity), enhanced cell–matrix interaction leading to better attachment (and sometimes alignment) of cells in/on 3D-bioprinted scaffolds, and improved cell maturation/differentiation. Moreover, the intrinsic properties of nanomaterials make them suitable for designing stimuli-responsive scaffolds, making them promising candidates for the next generation of bioinks for 3D-bioprinting. However, addressing potential concerns/setbacks associated with using nanofillers in 3D-bioprinting is essential to achieve the goal of clinical applications for 3D-bioprinted scaffolds and to enable wider uptake in the research community.

Foremost among these concerns is the critical necessity to ensure the biocompatibility of nanofillers. Some nanomaterials are highly biocompatible and safe, and some of have even been approved by the FDA. However, longer-term studies in larger animal models, may be warranted to fully assess the biocompatibility of nanofiller used for 3D-bioprinting. Moreover, the pharmacokinetics, toxicokinetics, metabolism, and biodegradation of 3D-bioprinted scaffolds containing these additives are not yet fully understood, but they are important to consider in almost all tissue engineering applications. It is crucial to consider the long-term stability of nanofiller-enhanced constructs. Changes in their material properties over time, such as degradation or loss of structural integrity, can significantly affect the functionality and viability of printed tissues. To address durability concerns, it is necessary to conduct extensive long-term stability studies and optimize the composition of nanofiller-enhanced constructs. One of the ways to improve stability is by incorporating biodegradable nanofillers with controlled degradation rates.

Ensuring a uniform dispersion of nanofillers in bioinks or printing materials is also a crucial aspect to consider as non-uniform distribution of these additives can lead to inconsistencies in the printed structure. This can significantly affect both the mechanical and biological properties of the printed structure. To tackle this issue, defined mixing protocol involving sonication or other methods for bioink homogenization, as well as nanofiller functionalization are being explored to enhance dispersion. Moreover, the use of advanced printing technologies, such as multi-material extrusion systems, can contribute to improving the uniformity of nanofiller distribution.

Researchers are continuously investigating new nanomaterials that are suitable for 3D-bioprinting. However, the range of biocompatible nanofillers available is currently limited. The ease of integrating these materials into printing processes also varies. Ongoing research in nanomaterial science aims to widen the selection of biocompatible nanofillers for 3D-bioprinting. The characterization of nanofillers, such as their shape, surface area, and topography, plays a critical role in determining the printability of bioink used for 3D-bioprinting as new nanofiller materials are identified. Despite the vast potential of this field, there are currently a lack of research studies that explore the impact of nanomaterial characterization on printability. Therefore, future research must investigate the influence of these parameters for nanomaterial fillers in bioinks to further advance the field of 3D-bioprinting. It is also important to note that the inclusion of novel nanofillers in bioinks may require adjustments in printing parameters, such as nozzle size, pressure, or temperature. To overcome the challenge of ensuring compatibility with existing 3D-bioprinters, collaborations between nanotechnology and the 3D-bioprinting experts will be necessary. Through such collaborations, specialized bioprinters optimized for nanofiller-enhanced bioinks can be developed. The printing parameters and nozzles can be customized and designed specifically for optimal performance. The impact of nanofillers on the rheological properties of bioinks is a crucial aspect that can significantly influence the printing resolution and accuracy. Achieving fine details in complex biological structures is a challenging task that can be addressed through the fine-tuning of bioink formulations and the optimization of printing conditions, such as temperature and pressure. Recent advancements in nozzle design and printing technologies hold promise for improving overall printing accuracy.

Finally, the widespread adoption of nanofiller-enhanced 3D-bioprinting will be tied to practical considerations of cost, scalability, and regulatory processes. If nanofillers are expensive or challenging to produce in large quantities, then it will be difficult to adopt them for 3D-bioprinting on a large scale. To address these concerns, it is necessary to explore cost-effective synthesis methods for nanofillers and to develop scalable production processes. The use of new nanomaterials in medical applications, especially in 3D-bioprinting for biomedical purposes, will require these materials to go through regulatory processes in various jurisdiction. Making sure that safety standards are complied with for clinical usage is a complicated process. Therefore, it is essential for researchers and regulatory organizations to establish standardized testing protocols for nanofiller-enhanced bioinks.

## Future Outlook

The future of nanofillers in 3D-bioprinting holds many possibilities, with potential advancements in several key areas. One of the potential avenues of exploration involves the introduction of smart nanofillers, which can respond to environmental stimuli or release drugs in a controlled manner. This innovation could lead to dynamic adjustments in printed structures, ultimately enhancing the functionality of 3D-bioprinted scaffolds.

The recent advancements in multi-material printing have provided significant opportunities for the creation of intricate and functional 3D-bioprinted tissues. This technology enables the simultaneous incorporation of nanofillers with diverse properties, leading to the development of complex structures with enhanced functionalities. Furthermore, the field is progressing toward personalized medicine applications, which ultimately may involve tailoring nanofiller-enhanced bioinks to meet patient-specific requirements. The progress in bioprinting with living cells such as stem cells is remarkable. This advancement has the potential to produce tissues that are more physiologically relevant and functional. As the field advances, efforts are being made to standardize bioprinting processes and materials, which will be important for gaining the appropriate regulatory approvals to move these technologies and materials to clinical settings.

It is expected that increased collaboration between researchers, clinicians, engineers, and industry partners will play a crucial role in advancing the field of nanofiller-enhanced 3D-bioprinting. This collaborative effort is vital for overcoming challenges and accelerating the translation of laboratory experimentation into practical medical applications. It is worth noting that this outlook for the future is speculative and subject to continuous progress and collaborative efforts within the scientific community. Regular updates and insights from the field will provide a more accurate understanding of the status and future directions of nanofiller applications in 3D-bioprinting.

## Data Availability

Not applicable.
